# Draft Genome Sequences of Nine Strains of *Ralstonia solanacearum* Differing in Virulence to Eggplant (*Solanum melongena*)

**DOI:** 10.1128/genomeA.01415-15

**Published:** 2016-01-28

**Authors:** Jérémy Guinard, Boris A. Vinatzer, Stéphane Poussier, Pierre Lefeuvre, Emmanuel Wicker

**Affiliations:** a Université de la Réunion, UMR 53 Peuplements Végétaux et Bioagresseurs en Milieu Tropical (PVBMT), Saint Denis, Réunion Island, France; b CIRAD, UMR 53 Peuplements Végétaux et Bioagresseurs en Milieu Tropical (PVBMT), Pôle de Protection des Plantes, Saint Pierre, Réunion Island, France; c Department of Plant Pathology, Physiology, and Weed Science, Virginia Tech, Latham Hall, Blacksburg, Virginia, USA

## Abstract

*Ralstonia solanacearum* displays variability in its virulence to solanaceous crops. We report here the draft genome sequences of eight phylotype I strains and one phylotype III strain differing in virulence to the resistant eggplant genotype AG91-25. These data will allow the identification of virulence- and avirulence-related genes.

## GENOME ANNOUNCEMENT

*Ralstonia solanacearum*, the causal agent of bacterial wilt, is one of the most harmful plant-pathogenic bacteria worldwide (1), causing tremendous yield losses within *Solanaceae* and *Musaceae* (2, 3). The *R. solanacearum* species complex (RSSC) is composed of four phylotypes (I, II, III, and IV). Among these, the pandemic phylotype I displays population structure (demographic expansion) and genomic features (recombination traces) probably at the origin of its high evolutionary potential (4), allowing it to adapt to new host species or resistant accessions of a host species. Among *Solanaceae*, eggplant is the crop species that carries the highest levels of resistance to *R. solanacearum*. So far, AG91-25 is the first eggplant genotype whose bacterial wilt resistance has been genetically mapped and associated with one major resistance gene (*ers1*) (5). Some *R. solanacearum* strains are totally controlled by AG91-25, while other strains are able to overcome its resistance (6, 7). To investigate the molecular bases driving these virulence differences, we sequenced nine strains of *R. solanacearum* that displayed different virulence patterns on AG91-25 (virulent, avirulent, and provoking latent infection) (J. Guinard, unpublished data; and 6–8), paying particular attention to differences in the type III effector gene (T3E) content between strains. Of these nine strains, eight are of phylotype I and originate from Asia (RUN0969 and RUN0157), Africa (RUN0215 and RUN1744), the Indian Ocean (RUN3012, RUN3013, and RUN3014), and South America (RUN1985), whereas RUN0039 belongs to phylotype III and originates from West Africa.

Genomic DNA was extracted using the Wizard genomic DNA purification kit (Promega). The strains were sequenced to a depth of ~100× using the Illumina HiSeq 2500 technology (150-base paired-end reads with an average insert size of 450 bp). The reads were quality trimmed using HTQC (9) before being assembled using CLC Genomics Workbench version 7.0.3 (CLC bio, Aarhus, Denmark). Genome assemblies were annotated automatically using the Web interface of the MicroScope platform (10). The nine genomes consist of 266 to 728 contigs. The genome sizes of the phylotype I strains range from 5.57 to 5.84 Mb, while the phylotype III genome is shorter, at 5.42 Mb. The overall G+C contents range from 66.86 to 67.05%. The draft genomes contain between 5,198 and 5,426 coding sequences, with 48 to 52 tRNA genes and 2 to 3 complete rRNA loci.

The genomes were submitted to the *Ralstonia* T3E website (https://iant.toulouse.inra.fr/bacteria/annotation/site/prj/T3Ev2/) (11) for T3E identification and *rip*-based annotation. The phylotype I strains contain 67 to 73 effector genes and two to seven pseudogenes, which are in the range of those in the reference phylotype I genome GMI1000 (11). Conversely, the phylotype III RUN39 contains only 55 T3Es and seven pseudogenes, which constitutes a T3E repertoire smaller than that of the phylotype I and phylotype III reference strain CMR15 (12). We did not find a clear association between T3E repertoire and virulence on AG91-25. However, the prevalence of the genes *ripAX2*, *ripA1*, *ripC2*, *ripE2*, *ripS6*, and *ripP2* was different between avirulent and virulent strains.

### Nucleotide sequence accession numbers.

The nine genome sequences have been deposited in the European Nucleotide Archive (ENA) under the study accession number PRJEB11298. The genome accession numbers are summarized in Table 1.

**TABLE 1:** Characteristics of the nine *R. solanacearum* strains

Strain	Genome	Alternative name	Accession no.	Phylotype	Sequevar^a^	*egl*-ST^b^	*mutS*-ST^b^	Host of isolation	Yr of isolation	Country	Pathoprofile^c^	Virulence on E6^d^	No. of contigs	Genome size (bp)
RUN0039	RUN39	CFBP3059	LN899819	III	23	002	005	*S. melongena*	1990	Burkina Faso	B	4	389	5,416,545
RUN0215	RUN215	CFBP7058	LN899820	I	13	019	030	*Solanum nigrum*	2005	Cameroon	A	2	403	5,839,544
RUN0157	PSS4		LN899821	I	15	015	028	*Solanum lycopersicum*	1988	Taiwan	E	4	374	5,680,609
RUN3013	RD1301	RD13.01	LN899822	I	31	043	022	*S. melongena* cv. E8	2012	Réunion Island		1	728	5,781,097
RUN1744	RUN1744	CIV 23	LN899823	I	31	043	022	*S. melongena*	2010	Ivory Coast	F	5	318	5,684,645
RUN1985	RUN1985	CIR011-208	LN899824	I	17	030	022	*S. melongena*	2011	French Guiana		5	266	5,788,639
RUN3012	TD1301	TD13.01	LN899825	I	31	043	022	*S. melongena* cv. E8 (susceptible)	2012	Réunion Island		2	511	5,711,184
RUN3014	TF3108	TF31.08	LN899826	I	31	043	022	Latent infections on *S. melongena* cv. E6 (resistant)	2012	Réunion Island		2	309	5,687,005
RUN0969	TO10		LN899827	I	47	050	022	*S. lycopersicum*	2003	Thailand		4	347	5,566,589

a Sequevars cluster strains whose *egl* partial sequence share ≥99% nucleotide identity (8).

b *egl* and *mutS* sequence types (ST), as presented in N’Guessan et al. (7) and Deberdt et al. (8).

c Pathoprofile is the pattern of interactions to tomato, eggplant, and pepper, as detailed by Lebeau et al. (6).

d The number assigned to each strain × accession combination represents the phenotype score, defined by the combination of final wilting incidence and colonization index and calculated as defined by Lebeau et al. (6): 1, highly resistant; 2, moderately resistant; 3.1, partially resistant; 3.2, latent infection; 4, moderately susceptible; 5, highly susceptible.
